# Antifungal Activity of Avocado Seed Recombinant GASA/Snakin PaSn

**DOI:** 10.3390/antibiotics11111558

**Published:** 2022-11-05

**Authors:** Marco Antonio Hernández-Martínez, Luis María Suárez-Rodríguez, Joel Edmundo López-Meza, Alejandra Ochoa-Zarzosa, Rafael Salgado-Garciglia, Silvia Patricia Fernández-Pavia, Rodolfo López-Gómez

**Affiliations:** 1 Instituto de Investigaciones Químico-Biológicas, Universidad Michoacana de San Nicolás de Hidalgo, Morelia 58030, Mexico; 2 Centro Multidisciplinario de Estudios en Biotecnología, Universidad Michoacana de San Nicolás de Hidalgo, Tarimbaro 58893, Mexico; 3 Instituto de Investigaciones Agropecuarias y Forestales, Universidad Michoacana de San Nicolás de Hidalgo, Tarimbaro 58893, Mexico

**Keywords:** avocado, antifungal, antimicrobial peptides, snakin

## Abstract

The avocado fruit (*Persea americana*) has become a significant fruit in the human diet for its nutritional properties. However, the seed is a source of bioactive molecules and has been poorly utilized. Previously, we reported that the PaSn gene is expressed in the avocado seeds, a cysteine-rich antimicrobial peptide (GASA/Snakin), and demonstrated its antibacterial activity. In this work, we report the recombinant production of PaSn in the *Escherichia coli* system and evaluate its antifungal activity against plant and human pathogen fungi. The recombinant peptide showed antifungal activity at 200 μg/mL against phytopathogens *Colletotrichum gloeosporioides* and *Fusarium oxysporum* and human pathogens *Candida albicans* and *C. glabrata*. Our results demonstrate the usefulness of a prokaryotic expression system for avocado antimicrobial peptide production. In conclusion, the snakin PaSn could be helpful in the control of postharvest avocado and other fruits’ fungal diseases and human fungal pathogens.

## 1. Introduction

Avocado fruit has become very important in the human diet. The seeds are the inedible part and are a source for searching for bioactive metabolites. Various extracts and metabolites from avocado seeds have been reported to have antimicrobial activities [1,2,3,4]. Gibberellin acid-stimulated Arabidopsis (GASA) proteins, previously named snakins, are plant cysteine-rich antimicrobial peptides (AMPs) isolated first from potatoes [5]. These peptides have been identified in different plants [6,7] and have antimicrobial activity as part of the plant defense system; they also take part in diverse plant developmental processes such as elongation and cell division, as well as flowering [6].

Avocado snakin (PaSn) is an abundant gene expressed during seed development that codes for a GASA/Snakin cysteine-rich antimicrobial peptide with antibacterial activity [7,8,9]. The PaSn gene was isolated from a cDNA library of the native Mexican avocado seed. This clone contains a coding sequence of 318 bp. It encodes a predicted 105 amino acid peptides, which comprises a 26 amino acid signal peptide and a 79 amino acid mature peptide with 12 conserved cyateine residues characteristic of this type of AMPs. PaSn heterologously expressed in the bovine endothelial cell line BVE-E6E7 showed antibacterial activity against *E. coli* and *S. aureus* [7,9].

Phytopathogenic fungi cause significant losses in crops and are the primary cause of postharvest diseases of fruits and vegetables. For example, the fungus *Colletotrichum gloeosporioides* is the causal agent of anthracnose in avocados, which causes production losses near 20% [10]. Fruit production, such as strawberry, mango, citrus, chili, and papaya production, is also significantly affected. Another important phytopathogenic fungus is *Fusarium oxysporum*, which has a wide host range and is responsible for severe losses in crops such as tomato, cotton, and banana [11]. On the other hand, in recent years, there has been a rise in human fungal pathogen resistance against conventional antifungals. Candida species, such as *C. albicans*, stand out because they are the primary causative agents of nosocomial fungal infections [12]. Therefore, there is a need to find pathogen control alternatives.

This work describes the heterologous expression of the GASA/Snakin PaSn gene from avocado seed in *E. coli*. In addition, the antifungal activity of recombinant PaSn snakin was tested against two fungal phytopathogens and two human fungal pathogens. The results showed that this AMP peptide has antifungal activity.

## 2. Results

### 2.1. E. coli Avocado PaSn Recombinant Proteint Expression and Purification

**Figure 1:**
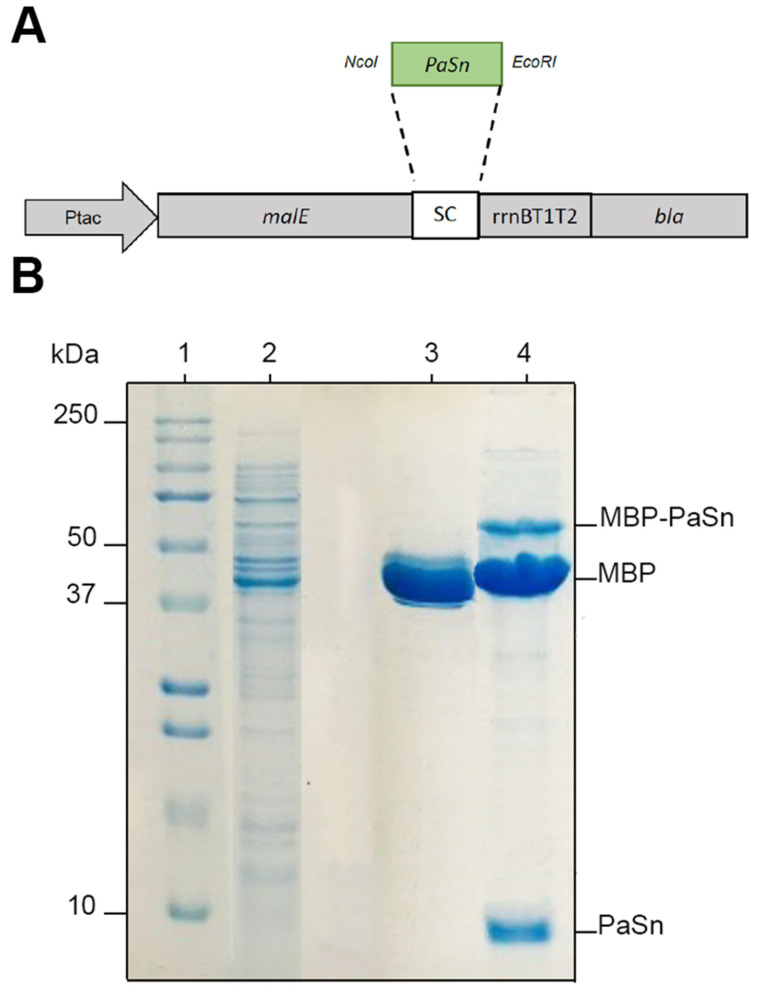
(**A**) Map of the construct pMALc5x-PaSn vector expression. (**B**) SDS-PAGE analysis of purified recombinant PaSn. Soluble proteins of different purification steps. Lane 1, low-molecular-weight marker. Lane 2, total bacterial proteins. Lane 3, proteins after affinity column. Lane 4, proteins after Factor Xa digestion.

PaSn was expressed as a fusion protein with MBP (Figure 1A). The calculated molecular mass for PaSn is 8 kDa and 44 kDa for MBP; thus, the estimated fusion protein was ~52 kDa. Bacteria were induced with IPTG to obtain the fusion protein. MBP-PaSn fusion protein ~45 kDa was detected by SDS-PAGE; this size was similar to the estimated (Figure 1B). Further, the MBP-PaSn fusion protein was purified and proteolytically digested with Xa factor. After digestion, two bands were detected in the gel, which correspond to the MBP (~45 kDa) and PaSn (~8 kDa) proteins (Figure 1B). The PaSn recombinant protein yield was 1106.6 µg/mL.

### 2.2. Identification of Heterologous Avocado PaSn Protein

In order to identify the ~8 kDa recombinant PaSn amino acid composition sequence, an SDS-PAGE purified band was digested with trypsin, and the resulting peptides were analyzed by MALDI-TOF mass spectrometry. We compared the pattern of peptide masses with those deduced from databases. A total of 79 peptide spectrum matches were obtained, which were grouped into seven unique peptides with a score of 210.28 using SEQUEST software. These seven peptides showed a high homology with a snakin from a *Persea americana* var. drymifolia protein noted in the uniprot database (Accession: L7WV37) (Table S1 Supplementary Material).

### 2.3. Growth Inhibitory Activity of PaSn against Phytopathogen Filamentous Fungi

**Figure 2:**
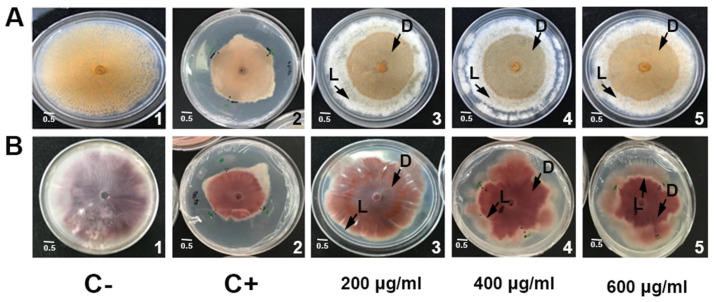
PaSn antifungal activity *in vitro* against phytopathogens using different peptide concentrations (**A**) *Colletotrichum gloeosporioides* (1–5), and (**B**) *Fusarium oxysporum* (1–5) mycelial growth inhibition. **1**: MBP, **2**: Commercial antifungal Tecto 60 (1 mg/mL), **3**: PaSn (200 μg/mL), **4**: PaSn (400 μg/mL), **5**: PaSn (600 μg/mL). Arrows: Mycelial differential growth **L**: Light zone, **D**: Dark. After 72 h incubation.

To determine whether the recombinant avocado PaSn has antifungal activity, we performed an inhibition in an *in vitro* agar diffusion assay against *C. gloeosporioides* and *F. oxysporum* fungi. We observed two growth zones differentiated by dark and light colors (Figure 2). In both fungal colonies, the appearance of mycelium with different colors (dark and light) suggests that avocado snakin affects mycelial development. These changes in mycelium color could be due to an alteration of the fungus sporulation. We observed this phenomenon in all PaSn concentrations used. For both phytopathogens, the snakin antifungal effect was similar to the commercial Tecto60 fungicide. Additionally, MBP does not show any antifungal activity, which supports that PaSn was responsible for the antifungal activity (Figure 2).

### 2.4. Permeabilization of Phytopathogen Membrane

**Figure 3:**
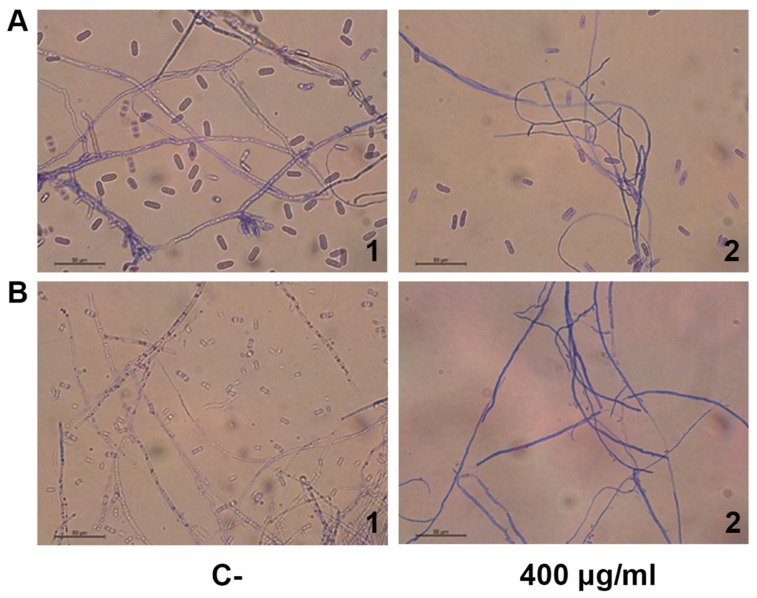
PaSn plasma membrane permeabilization. Optical micrographs 40X displaying staining mycelia with trypan blue 5%. (**A**) *Colletotrichum gloeosporioides* (1–2), and (**B**) *Fusarium oxysporum* (1–2) mycelial growth inhibition. **1**: MBP negative control, **2**: PaSn (400 μg/mL).

Trypan blue is a diazo dye commonly used to test cell viability because it can only infiltrate cells with a damaged membrane, whereas healthy cells will not be stained [13]. Mycelial samples from different colors of each fungus’s growth zone were tested *in vitro* with varying concentrations of recombinant PaSn and then stained with trypan blue and observed under the optical microscope. The mycelial PaSn-treated cells were stained blue compared to the control (Figure 3A,B), indicating that PaSn generates damage in the hyphal cell’s membrane. One interesting observation is that the fungal spores were scarce.

### 2.5. Phytopathogen Fungal Inhibition Spore Germination

**Figure 4:**
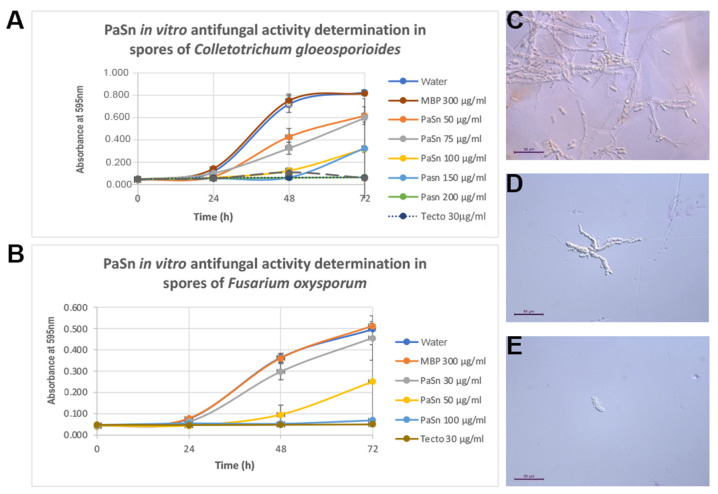
Phytopathogen spore germination. *In vitro* fungal phytopathogen spore germination was assessed using an MTT viability assay with different PaSn concentrations. (**A**) *Colletotrichum gloeosporioides*, and (**B**) *Fusarium oxysporum*. (**C**–**E**): *C. gloeosporioides* micrograph of spore germination alteration (**C**): Control negative, (**D**,**E**): PaSn 300 μg/mL. Data were analyzed by ANOVA one-way and post hoc Tukey’s test (*p* < 0.05).

The PaSn effect on spore germination was determined by MTT assay. We assessed the PaSn IC_50_ for each fungus by concentration-response curves (Figure 4A,B). The results showed that PaSn protein had an IC_50_ of 111 μg/mL for *C. gloeosporioides* and 59.8 μg/mL for *F. oxysporum*. These results indicate that the PaSn antifungal activity could vary according to phytopathogen species. The PaSn concentrations of 300 μg/mL and 100 μg/mL showed a similar effect to commercial fungicide on spore germination of *C. gloeosporioides* and *F. oxysporum*, respectively.

### 2.6. PaSn Activity against Human Pathogen Fungal

**Figure 5:**
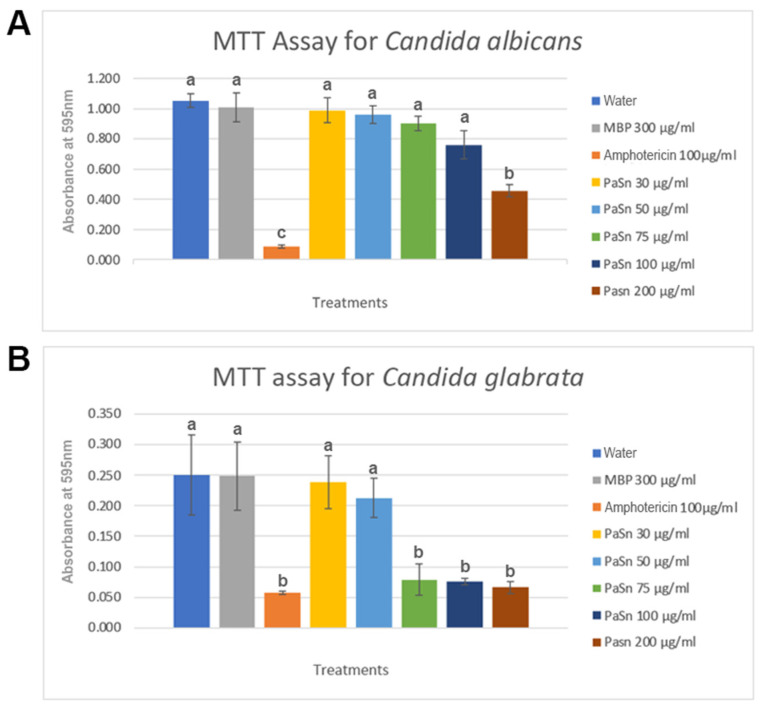
*In vitro* effects of avocado PaSn recombinant peptide in a human pathogen viability assay. MTT viability assay with different concentrations of PaSn. (**A**) *Candida albicans* (**B**) *Candida glabrata*. Each concentration was replicated in three independent experiments. There were significant differences between groups. Multiple group comparisons were tested using analysis of variance (ANOVA) in the SPSS software. Differences were defined as significant at *p* < 0.05. Different letters indicate a significant difference between groups, while the same letter indicates no difference.

*C. glabrata* and *C. albicans* were grown in microplates for 72 h with different concentrations of recombinant PaSn (0–200 μg/mL). For *C. albicans,* we observed an apparent reduction in viability at 200 μg/mL. We still did not observe a direct relationship between concentration and fungal viability. However, for *C. glabrata*, the activity of PaSn was different, as we observed a drastic decrease in the fungal viability at the concentration of 75 μg/mL, similar to the antifungal amphotericin used as a positive control (Figure 5A,B).

## 3. Discussion and Conclusions

The expression of avocado PaSn in the pMAL bacterial heterologous system was successful; we obtained an efficiency of purified recombinant protein of 1106.6 μg/mL (Figure 1). This value is similar to that for other plant snakin produced in heterologous systems [14]. Snakin genes from other plants have been expressed successfully using heterologous systems such as potatoes [15,16], tomatoes [17], and rice [14]. To assess the antifungal activities of recombinant PaSn, we performed several antifungal growth inhibition assays against avocado and human fungal pathogens. Avocado pathogens were susceptible to PaSn, and we differentiated two zones of mycelial growth (dark and light) at 200 μg/mL. These changes in mycelium color could be due to an alteration of the fungus sporulation. Accordingly, the light zones presented perforated mycelial (Figure 3) and scarce spore presence compared with the negative control. These results suggest that PaSn altered spore development in both phytopathogen fungal species (Figure 2, Figure 3 and Figure 4). One interesting observation was that avocado snakin produced an inhibitory growth effect similar to the commercial fungicide Tecto60. Avocado PaSn recombinant protein inhibited spore germination for both phytopathogens (Figure 4A,B). The fungal membrane permeabilization activity and spore germination inhibition have been reported for *C. coccoides* and *Botrytis cinerea* at 14 mM concentrations of potato snakin-1 [15]. These results are interesting and coincide with the report for another kind of AMP, olive defensin, which shows complete inhibition of spore germination and permeabilization of the membrane in ascomycete fungal pathogens [18]. This suggests a similar mode of action between cysteine-rich AMPs. Additionally, recombinant avocado GASA/Snakin showed a significative antifungal effect on the human fungal pathogens *C. glabrata* and *C. albicans*, both of which showed different sensibility to the avocado snakin (Figure 5A,B). These results suggest a difference in sensibility to snakin in plant and human pathogens. The different sensitivity presented by the different fungi to PaSn could be a consequence of the composition of the fungal cell wall that occurs during the stages of development and fungal species [18,19]. Also, it is known that the activity of these peptides depends on the correct formation of disulfide bridges. In this sense, we consider that the demonstration of antifungal activity is in line with the formation of disulfide bridges, since otherwise the biological activity would not have been presented. With the results of this work, we conclude that snakin PaSn from avocado had antifungal activity against phytopathogen and human fungi tested *in vitro*. Considering the previous results reported and this work, PaSn avocado snakin could be a good antimicrobial candidate. However, more studies are needed to determine the biotechnological potential of this AMP.

## 4. Materials and Methods

### 4.1. Fungal Strains

Two phytopathogen fungi were used: *Colletotrichum gloeosporioides* and *Fusarium oxysporum* were obtained from the Phytopathology Laboratory of the Universidad Michoacana de San Nicolás de Hidalgo. In addition, the human clinical isolate *Candida albicans* ATCC 10231 was used. Additionally, *C. glabrata* was kindly donated by Dr. Angel G. Alpuche-Solis (Instituto Potosino de Investigación Científica y Tecnológica A.C., San Luis Potosí, México).

### 4.2. Construction of Recombinant Plasmid, Expression, and Purification of Recombinant PaSn

The avocado PaSn cDNA (NCBI Accession No: KC012806.1) was obtained from the avocado seed cDNA library in the pTRIplEx vector (Takara Bio™) [7]. The pMAL-c5X vector (New England Biolabs, Beverly, MA, USA) was used for the bacterial expression of avocado snakin as a fusion protein with maltose binding protein (MBP). For this, we constructed the expression cassette pMal-PaSn (Figure 1A) and transformed it into *E. coli* ER2523 cells. Using the bioinformatic program UGENE, we designed primers for cloning the PaSn coding without the signal peptide sequence. Primers FW 5’- CAT GCC ATG GCA TGG TTT CAG TCT CAT TCG-3′ and RW 5′-CCG GAA TTC TTA AGG ACA TTT GCG TTT GTT CC-3′ were used for amplification of PaSn with *Taq* DNA polymerase recombinant (Invitrogen™ cat. no. 11615-036). PCR conditions were as follows: an initial 94 °C denaturation step for 2 min followed by denaturation for 15 s at 94 °C, annealing for 30 s at 63 °C, and extension for 30 s at 72 °C for 35 cycles, final extension 72 °C for 10 min. PCR products were directionally cloned into pMAL-c5X using *Nco*I and *Eco*RI restriction sites and transformed into *E. coli* ER2523. After PCR colony screening, the DNA sequences of the positive clones were confirmed by DNA Sanger sequencing.

To produce the recombinant PaSn, bacteria were induced with 0.3 mM IPTG at 37 °C for 6 h. Next, cells were harvested by centrifugation, resuspended in a column buffer, and sonicated. Cell debris was precipitated by centrifugation, and the supernatant with the fusion protein was recovered. The presence of MBP-PaSn fusion protein was evaluated by 10% SDS-PAGE. Protein purification and elution were made by affinity chromatography in ÄKTA-pure equipment and MBPTrap Hp (cat. no. 28-9187-78, GE Healthcare). Recombinant MBP-PaSn peptide was proteolytically cleaved with Factor Xa protease to release mature snakin (New England BioLabs cat. no. P8010S). The peptide digestion and size were verified by SDS-PAGE 10%. Furthermore, the peptide mix was dialyzed with a membrane tube (Sigma-Aldrich, St Louis, MO, USA cat. no. D7884) for 24 h. Finally, PaSn purification was conducted by molecular exclusion chromatography using a Bio-Gel P10 (Bio-Rad Laboratories, cat. no. 1504144); it was then resuspended in 20 mM Tris-HCl, 200 mM NaCl, 1 mM EDTA, pH 6.4, and stored at −4 °C until use. The protein concentration was determined according to the Bradford assay. Bovine serum albumin was used as standard [20]. In addition, before the assays, disulfide bonds were formed by air oxidation in 5% (*v*/*v*) aqueous dimethyl sulfoxide at a concentration of 500 μg/mL for 24 h at room temperature [21].

### 4.3. Identification of Recombinant Avocado PaSn by Mass Spectrometry

The purity of eluted PaSn was evaluated by separating the peptide on a 15% (*w*/*v*) Tris-Glycine gel; after separation, the peptide band was visualized by Coomassie staining. Next, the band was cut, washed, and digested overnight with trypsin. The resulting peptides were extracted from gel pieces with formic acid [22]. Further, tryptic peptides were applied to a nanoAcquity nanoflow LC system (Waters Corp. Milford, MA, USA) coupled to a linear ion trap LTQ Velos mass spectrometer (Thermo Fisher Scientific, Bremen, Germany) equipped with an electrospray ion source. Finally, we used the peptide sequences and the SEQUEST software with the UniprotKB database for protein identity.

### 4.4. In vitro Antifungal Activity against Phytopathogens and Membrane Permeabilization

The *in vitro* antifungal activity of recombinant PaSn was assayed by mycelial growth in Petri dishes with a modification of the agar well diffusion technique [23]. The fungi were grown in PDA solid medium for 72 h at 28 °C under dark conditions. Furthermore, we took 2 cm blocks from the growing zone and put them in the center of Petri dishes with different concentrations of recombinant PaSn (200, 400, and 600 μg/mL of protein) in the perimeter of the Petri dish. The commercial antifungal Tecto 60 (thiabendazole) (1 mg/mL) was used as a positive control. The membrane permeabilization of fungal cells was analyzed using light microscopy to visualize the influx of Trypan Blue dye.

### 4.5. Fungal Spore Germination Inhibition Assay

Spore suspensions (2 × 10^3^ spores) of *C. gloeosporioides* and *F. oxysporum* were sowed in 100 μL of PDY medium in 96-well flat-bottom plates. To obtain half maximal inhibitory concentration (IC_50_) for each fungus, several concentrations of recombinant snakin were used: 50, 75, 100, 150, 200, and 300 μg/mL for *C. gloeosporioides* and 30, 50, and 100 μg/mL for *F. oxysporum*. Commercial fungicide Tecto60 was used as the positive control (30 μg/mL) and water and MBP (300 μg/mL) were used as negative controls. Plates were incubated at 28 °C for three days, and each 24 h, we measured the absorbance at 595 nm in a BioTek™ Epoch Microplate Spectrophotometer System at 24, 48, and 72 h. We used the average absorbance data of three replicas of each treatment to generate a concentration–response curve. Finally, the inhibitory activity on fungal spore germination was visualized by light microscopy at 72 h incubation.

### 4.6. Human Pathogen Fungal Growth Inhibition Assay by MTT

*C. albicans and C. glabrata* cells (100,000 CFU) were inoculated in 200 μL of PDY growth medium. First, cells were incubated with different concentrations of PaSn recombinant peptide (30, 50, 75, 100, and 200 μg/mL) for 24 h at 37 °C in a 96-well flat-bottom plate. Next, 10 μL MTT (5 mg/mL) was added, and plates were left for 4 h at 37 °C. The reaction was stopped with 100 μL isopropanol/HCl 1M (19:1). The absorbance was measured in a microplate reader at 595 nm. The positive control was amphotericin 100 μg/mL. PDY was used as a negative control, with MBP (300 μg/mL) as an additional control. All the samples were triplicated, and the absorbance changes were averaged.

### 4.7. Statistical Analysis

In this study, data were analyzed by ANOVA and Tukey’s statistical analysis using PAST v2.17c software.

## Data Availability

Not applicable.

## Supplementary Materials

The following supporting information can be downloaded at: https://www.mdpi.com/article/10.3390/antibiotics11111558/s1, Table S1: Recombinant purified avocado PaSn amino acid sequences obtained by MALDI-TOF and their corresponding association with predicted sequences from the databases^a^.
