# Supplementary material for: Antifungal Activity of Avocado Seed Recombinant GASA/Snakin PaSn
Source: Antibiotics (Basel). 2022 Nov 5;11(11):1558. doi: 10.3390/antibiotics11111558 (PMC9686948; doi:10.3390/antibiotics11111558)
Supplement: Supplementary file 1 [file antibiotics-11-01558-s001.zip › antibiotics-1992027-supplementary.pdf]

Supplementary material

Table S1. Recombinant purified avocado PaSn amino acid sequences obtained by MALDI-TOF and their corresponding association with predicted sequences from the databases[a].

Number	Sequence	Range	#PSMs	XCorr
1	NLLQQIDCGTSCSAR	40-54	32	5.35
2	CNCVPSGTAGNLDECPCYANMTTHGNK	75-101	3	4.54
3	NLLQQIDCGTSCSAR	40-54	2	3.55
4	RACGTCCAR	66-74	8	3.07
5	NLLQQIDCGTSCSARCR	40-56	1	3.00
6	ACGTCCAR	67-74	13	2.12
7	CNCVPSGTAGNLDECPCYANMTTHGNKR	75-102	1	1.83
8	LSSRPR	57-62	19	1.82

[a]The peptides were alkylated with iodoacetamide. The final score=210.28 and sequence coverage=57.14 were detected.
